# A Spanish clinical perspective on national regularization of allergen immunotherapy products 

**DOI:** 10.5414/ALX400671

**Published:** 2026-09-25

**Authors:** Ruperto González-Pérez, Paloma Poza-Guedes, Inmaculada Sánchez-Machín

**Affiliations:** 1 Allergy Department,; 2 Severe Asthma Unit, Allergy Department, Hospital Universitario de Canarias,; 3 Instituto de Investigación Sanitaria de Canarias (IISC), and; 4 Allergen Immunotherapy Unit, Allergy Department, Hospital Universitario de Canarias, Tenerife, Spain

**Keywords:** allergen immunotherapy, regulatory framework, named patient products, precision medicine, marketing authorization

Sir, – The article by Mahler et al. [1], “*Therapy Allergen Ordinance (TAO) – The final stretch*”, provides a timely and thorough overview of the German process for converting named patient products (NPPs) for allergen immunotherapy (AIT) into fully authorized medicinal products. Their experience is of particular interest in Spain, where a new national framework for allergen-based medicines is being implemented and has generated considerable concern among clinicians and manufacturers. 

In Spain, recent measures introduced by the National Medicines Agency (AEMPS) require industrially manufactured allergen-based medicines – both therapeutic and diagnostic preparations – to obtain marketing authorization or follow a defined registration pathway [2]. At the same time, individualized NPPs are now formally limited to exceptional cases where no authorized alternative exists and must comply with strict notification and manufacturing requirements [2]. As a result, many long-standing formulations will either need to be supported by complete clinical data or leave the market. While this mirrors the overarching goals of the German transition, structural distinctions exist: the German TAO selectively targeted only high-prevalence allergens (leaving niche allergens accessible via the NPP route) and left in vivo diagnostics unaffected, whereas the Spanish framework encompasses all industrially manufactured preparations under its mandate [1, 2]. Concretely, the Spanish pathway establishes a progressive 5-step procedure, with initial marketing authorization application (MAA) deadlines for various allergen groups extending up to December 2030 [2]. Crucially, these initial MAAs do not require an immediately complete dossier; instead, manufacturers are granted an ongoing, step-by-step update pathway [2]. This operational characteristic, shared with the German TAO experience, provides essential time to gather quality and clinical evidence while proactively safeguarding immediate product availability. 

The German TAO experience illustrates how such reforms may significantly reduce the number of marketable AIT products while raising the overall level of evidence. For Spain, which has historically relied on an especially broad portfolio of individualized preparations, this presents a real risk of contraction – particularly for niche allergens, complex mixtures for polysensitized patients, and products intended for small patient subgroups. From a clinical standpoint, diminished product diversity may hinder the tailoring of AIT and could inadvertently create inequities between patients sensitized to frequent allergens and those with less common profiles [3, 4, 5, 6]. However, this clinical concern must be framed within current state-of-the-art standards. According to the EMA *Guideline on Allergen Products: Production and Quality Issues*, complex allergen mixtures are strictly restricted to avoid proteolytic degradation, and the mixing of seasonal and perennial allergens or distinct venom genera is discouraged unless explicitly justified [7]. Thus, the expected market contraction also serves to align clinical practice with modern, evidence-based quality constraints. Furthermore, while these patient inequities are a known phenomenon across all low-prevalence medical conditions due to the inherent difficulties of trial recruitment, important regulatory mechanisms have emerged to mitigate this gap. Specifically, the newly issued EMA *Guideline on allergen products development for immunotherapy and allergy diagnosis in moderate to low-sized study populations* introduces adaptive trial designs and modified clinical requirements tailored precisely to low-prevalence allergens and smaller manufacturers, offering a vital safety net for product diversity [8]. 

A second shared challenge concerns the proportionality of evidence requirements. Under TAO, marketing authorization is granted only when robust evidence of quality, safety, and efficacy is provided, often through dose-finding and pivotal phase II/III studies demonstrating both statistical significance and clinically relevant benefit. The Spanish framework likewise calls for full quality and clinical documentation for industrially manufactured allergen-based medicines and cites the EMA guideline on clinical development of specific immunotherapy products as the scientific reference [9]. While appropriate for high-prevalence allergens such as house dust mites and grasses, generating such data for low-prevalence allergens or long-used preparations produced by smaller manufacturers may be difficult and could result in unintended product losses. Importantly, the burden of generating clinical data is significantly alleviated by the concept of “homologous groups” and cross-reacting allergen extrapolation, principles that apply to both the German and Spanish pathways under EMA guidelines [7]. Efficacy and safety data can be extrapolated from a representative source allergen to other members of the same homologous group, meaning distinct clinical trials are not mandated for every single individual formulation. 

A third issue, common to both systems, relates to treatment continuity. In Germany, rejection of a marketing authorization under TAO immediately ends marketability, although dispensed vials can generally be used until their expiry date. Spanish documents similarly indicate that products not entering or not completing the regularization pathway may no longer place new batches on the market once deadlines or decisions are reached. For allergists, changing AIT in stable patients within limited timeframes may be clinically and ethically challenging and may negatively affect adherence and patient confidence [10]. Importantly, AEMPS presents the Spanish initiative as a national solution developed within the broader EU and EMA framework, not as a direct transposition of the German TAO. This initiative was built directly upon the *Coordinating Group for Mutual Recognition and Decentralised Procedures – Human (CMDh) recommendations on common regulatory approaches for allergen products*, which Spain has followed to shape its national pathway [11]. This distinction offers an opportunity to incorporate lessons from Germany while designing a more balanced system for Spain – one that combines rigorous evidence requirements with risk-adapted approaches and strong safeguards to preserve treatment continuity. 

Given these shared challenges and national specificities, we believe that continued constructive discussion on how different regulatory strategies affect real-world AIT availability and patient care would be of substantial value to practicing allergists. To successfully balance the push for robust evidence-based precision medicine with the clinical necessity of maintaining a diverse allergen panel, we recommend three specific future pillars: first, competent authorities should fully exploit the flexibility of the new EMA/161669/2025 guideline, accepting real-world evidence and registry data to supplement small-population trials for niche allergens; second, the definition of homologous groups should be dynamically updated to maximize scientifically sound clinical data extrapolation; and third, formal communication channels between regulatory bodies, manufacturers, and professional allergy societies must be mandated to proactively identify critical product gaps before deadlines expire, ensuring that high-evidence models do not inadvertently compromise individualized patient care. 

## Authors’ contributions 

Conceptualization: RG-P and PP-G; methodology: RG-P and PP-G; validation: IS-MM, SE; formal analysis: RG-P, PP-G, and IS-M; writing—original draft preparation: RG-P; writing—review and editing: PP-G, and IS-M; supervision: IS-M; project administration: RG-P and PP-G. All authors approved the final version of the manuscript. 

## Funding 

The authors received no financial support for the research, authorship, or publication of this article. 

## Conflict of interest 

Authors (RG-P, PP-G, and IS-M) declare that this research was conducted in the absence of any commercial or financial relationships that could be construed as a potential conflict of interest. 
